# Adolescents' depressive moods and parents' family-work interaction

**DOI:** 10.3389/fpubh.2022.975935

**Published:** 2023-01-06

**Authors:** Li Lu

**Affiliations:** School of Psychological and Cognitive Sciences and Beijing Key Laboratory of Behavior and Mental Health, Peking University, Beijing, China

**Keywords:** adolescent depressive moods, family-work conflict, work engagement, performance-avoidance, child-parent level

## Abstract

**Introduction:**

For working parents with dependent children, parenthood is essential to their “life” component, which could profoundly influence their work experiences. Since depressive moods rise sharply in adolescence, this study aims to investigate the relationship between children's depressive moods and parental family-work interaction. Integrating the literature on emotions and family-work interaction, I propose that adolescents' depressive moods (over the past 2 weeks) decrease parents' work engagement *via* increased parents' family-work conflict. Further, I hypothesize that adolescent performance-avoidance, a key trait related to adolescents' long-term emotional experiences, moderates the indirect relationship.

**Methods:**

Using a multiple-source, time-lagged design, I tested hypotheses using data collected from 468 adolescent-parent dyadic from China.

**Results:**

I found that adolescents' depressive moods relate negatively to their parents' work engagement *via* increased parents' family-work conflict when adolescents have low levels of performance-avoidance. When an adolescent has a high level of performance-avoidance, parents show a relatively higher degree of family-work conflict and lower work engagement regardless of adolescents' depressive moods.

**Discussion:**

I discuss the theoretical and practical implications for employee family-work interaction and work engagement.

## 1. Introduction

Clinical depression in adolescents is increasingly becoming a global problem (1–3). According to the National Institute of Mental Health (4), 12.5% of 12–17-year-olds in the United States have experienced symptoms of a major depressive episode for up to 12 months, and nearly a fifth of them have experienced a major depressive episode in the middle and later stages of their adolescence. While a prevailing view is that parents are in the position to exert power and influence their offspring's mental health [e.g., (5–8)], there is a growing consensus that children's mental health problems also affect parental mental health (9–12). Such that depression in adolescents represents a potential risk factor for the development of parents' mental health difficulties (13, 14). Notably, these studies have focused mainly on long-term depression and clinical samples.

Depressive mood, like clinical depression, increases steeply during adolescence (15–18). However, it is shorter in duration, less severe in symptomatology, and more widespread in the general population than clinical depression. Kessler et al. (19) reported that approximately 20 to 50% of adolescents reported significant subsyndromal levels of depression from 1 week to 6 months. Depressive moods in adolescents have destructive consequences: When depressed, adolescents constantly experience high levels of negative and low levels of positive emotions (20).

Thus, short-term depressive moods in adolescents might have essential yet neglected negative impacts on a substantial proportion of parents. The present study aims to investigate the relationship between adolescent depressive moods (over the past 2 weeks) and parents' family-work interaction. As the structure of society changes, dual-earner families are becoming more prevalent. This means that many of today's employees need to juggle their family roles with their work roles, and their familial and occupational well-being is reciprocal in nature [e.g., (21)]. On the one hand, working parents have aspirations to nurture and care for their dependent children (22). They take caregiver responsibilities to seek enhancement of their dependent children's well-being (23). Participating in family and work roles simultaneously, adolescent children's depressive moods might potentially influence working parents' work experiences. On the other hand, children's depressive moods might influence a parent's outcomes accumulatively [e.g., (24)]. Such that despite children's long-term clinical depression affects parental mental health difficulties (13, 14), children's momentary affects only have insignificant impacts on parental experiences (25–27). Therefore, it is also crucial to investigate the magnitude of negative organizational consequences caused by adolescents' short-term depressive moods and parental family-work conflict.

I propose that adolescents' short-term depressive moods (over the last 2 weeks) decrease parents' work engagement by elevating parents' family-work conflict. Depression in adolescence could be characterized as mood symptoms (20). Emotional experiences can transfer from one family member to another, leading to family-work interaction (26, 28, 29). Generally speaking, emotional transmission research conducted on the parent-child level considers that parents' emotions are likely to influence their children, but not the other way around (25–27). However, most researchers focused on the immediate effect of momentary affect transmission and ignored the potential impact of children's persistent, state-based emotional experiences on parents' family-work interaction. Adolescents' depressive moods often persist for weeks (19). When depressed, adolescent children experience increased negative moods and decreased positive moods (20). It might cause stressful parent-child events, leading to increased family-work conflicts and hindering parental work engagement.

Moreover, it is well demonstrated that individuals' state-based mood experiences and trait-based affect might jointly influence family members' family-work interaction (30). I propose that adolescent performance-avoidance orientation moderates the relationship between adolescent depressive moods and parent family-work conflict. Performance-avoidance orientation is the most negative orientation in achievement goals which constantly shapes adolescent children's moods experiences and causes parent-child stressful events. Adolescents with high performance-avoidance orientation always try to avoid performing worse than others (31). They tend to exhibit more negative emotions and encounter more stressful events (32). These may cause parents to constantly experience a high level of family-work conflict even when adolescent children have low depressive moods. Such that the positive effect of adolescent depressive moods on parents' family-work conflict is stronger when adolescent performance-avoidance is low than when it is high.

This research has three contributions to the study of emotions and family-work interaction at the child-parent level. First, I focus on the potential effect of children's persistent, state-based negative moods (depressive moods over the last 2 weeks) on parents' family-work interaction and point out the new essential factors that affect employees' family-work conflict. Since 20–50% of adolescent parents might be endangered by their children's depressive moods once in a while, it is of great practical significance to pay attention to and intervene in this phenomenon. Second, I point out that adolescent trait-based affect might cause potential and long-term influence on parent family-work interaction (inter-domain, inter-individual). Thirdly, I discuss how children's performance-avoidance orientation (trait-based emotion experiences) and children's depressive moods (persistent stated-based emotional experiences) jointly affect parents' family-work conflict and work engagement, which provides a new way to explore the relationships among individual traits, emotional experiences and family-work interaction in the dyadic level.

### 1.1. Theory and hypothesis development

Adolescence is a peak period for the onset of depressive moods (15–18). Adolescents encounter conflicting expectations from school, family, and society over the period. Stressful events (such as failure of academic tasks, interpersonal rejection, criticism, parent-child conflict, or lack of social support, *etc*.) may elevate depressive moods in adolescents. Nearly a fifth of adolescents have experienced diagnosable depression before age 18 (33, 34). Approximately 20 to 50% of adolescents reported significant subsyndromal levels of depression over a period from 1 week to 6 months (19). When feeling depressed, adolescents are usually distracted, feeling meaningless, prone to change of diet preference, or even have sleep problems. Emotionally, they are more irritable, depressed, facing anhedonia and loss of pleasure (20).

A growing body of evidence has emerged over recent decades suggesting that children's depression is related to their parents', particularly their mothers', clinical well-being (13, 14, 35, 36). Although less destructive than clinical diagnostic depression, depressive moods are more common in normal adolescents [20 to 50%, (19)]. It is crucial to investigate the consequences on parents' experiences of family-work conflicts during the period and subsequent parental family-work interaction.

### 1.2. Adolescents' depressive moods and parents' family-work conflicts

The intersection of work life and family life has been studied for decades [e.g., (21, 37–42)]. In broad terms, work-family interaction refers to experiences in the family (work) domain that impact experiences in the work (family) domain. Scholars recognize that the work-family interface has both positive and negative sides (41, 43–46). In particular, the negative work-family interface refers to a specific form of inter-role conflict where the obligations associated with family and work roles are mutually incompatible and where playing one role make it more difficult or stressful to play the other [e.g., (47–50)].

Affect play a key role in the family-work interface (26, 30). Adolescents' depressive moods often last for weeks (19), which might increase working parents' family-work conflict. One family member's emotional experiences in the family domain might influence another family member's family-work interaction (41). The parent-child level is one of the three crucial observation levels in the field of emotion and family-work interaction (30). However, compared with spouse-spouse and leader-subordinate levels, relatively fewer researches have focused on the relationship between moods and family-work interaction at the child-parent level. Previous studies suggest that children's momentary affects are less likely to influence parents' experiences (25–27).

Nevertheless, I suggest that adolescent children's depressive moods (over the last 2 weeks) increase parents' family-work conflict. Depressed adolescents are irritable, depressed, and anhedonic (39). Compared to momentary affects, depressive moods are recurrent in adolescence and often persist over a period of time (19). In this case, adolescent depressive moods may gradually and imperceptibly affect parents' family lives. Since parents pay more attention to fulfilling the family role's requirements, it becomes more challenging for them to put related resources into the work role (39, 40, 51).

Moreover, youth depressive moods may accompany a series of parent-child relational stressful events (such as negative communication, suicidal tendencies, *etc*.), which may also increase parents' family-work conflict. When feeling depressed, adolescents constantly hold negative attitudes and behave unhealthily; they might have low self-esteem, low life satisfaction, sleep problems, and low academic performance (1, 20). In the process of coping with depressed adolescents, parents are advocated to alleviate their children's depressive moods by changing their parenting style, avoiding negative interaction, giving interpersonal support, and improving relationship satisfaction (52–57). These behaviors may overwhelm parents with more pressures from the family field and force them to expend extra efforts to complete their family roles so that their resources at work are constrained (28, 58). So, children's depressive moods may improve parents' family-work conflict level through stress transfer.

*Hypothesis 1: Adolescents' depressive moods (over the last two weeks) related positively to parents' family-work conflicts*.

### 1.3. Adolescents' performance-avoidance orientation as a boundary condition

Achievement goal, also known as goal orientation, has been proven to be a particularly robust concept for predicting adolescents' motivation, emotions, and behaviors (59). In particular, performance-avoidance orientation leads to the most negative consequences compared to the other three types of goal orientations (32). Adolescents with high performance-avoidance may adopt more self-handicapping strategies (60) which leads to multiple maladaptive outcomes [for meta-analysis, see (59, 61–65)]. In particular, performance-avoidance orientation positively predicted negative emotions and negatively predicted performance attachment and task performance.

It is well demonstrated that state-based and trait-based emotional experiences jointly influence individuals' family-work interaction (30). I propose that performance-avoidance orientation moderates the relationship between adolescents' depressive moods and parental family-work conflict. Adolescents with a high level of performance avoidance orientation might constantly experience high levels of negative emotions leading to more stressful events, leading working parents to continually invest resources in the family domain and experience high levels of family-work conflict regardless of children's state-based emotional experiences. So, I propose that adolescent children's depressive moods related more positively to parents' family-work conflict when adolescents' performance-avoidance is low than when it is high.

*Hypothesis 2: Adolescents' performance-avoidance orientation negatively moderates the relationships between adolescents' depressive moods (over the last 2 weeks) and parents' family-work conflicts, such that the positive relationships are stronger when performance-avoidance is low than when performance-avoidance is high*.

### 1.4. Linking family-work conflicts to work engagement

Work engagement is an indicator of positive occupational well-being. I further propose that parental family-work conflict relates negatively to work engagement. When parents have to invest more energy in fulfilling the family role, they have less energy to fulfill their work role (30, 39, 58, 66, 67). Parents might find it challenging to maintain a positive, fulfilling, work-related state of mind that is characterized by vigor, dedication, and absorption (68, 69); further hindering parents' work engagement (70–72).

*Hypothesis 3: Parents' family-work conflict relates negatively to parents' work engagement*.

### 1.5. Integrated model

**Figure 1:**
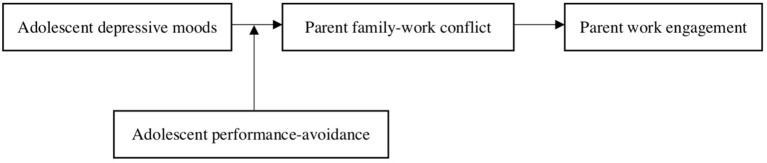
Theoretical model of adolescents' depressive moods and parents' work engagement.

To integrate these relationships, I propose a moderated mediation model in which adolescents' performance-avoidance orientation moderates the indirect negative relationship between adolescents' depressive moods and parents' work engagement *via* increased family-work conflict. That is, when adolescents' performance-avoidance is low, the indirect relationship between adolescents' depressive moods and their parents' work engagement *via* family-work conflict is stronger than when adolescents' performance-avoidance is high. For the theoretical model, see Figure 1.

*Hypothesis 4: Adolescents' performance-avoidance orientation negatively moderates the indirect relationship between adolescents' depressive moods (over last 2 weeks) and parents' work engagement via increased parents' family-work conflict, such that positive indirect relationships become stronger when performance-avoidance is low than when performance-avoidance is high*.

## 2. Method

### 2.1. Sample and procedure

I focus on a general population sample rather than a clinical sample. Participants were recruited from a local senior high school in China. The data collection was conducted with the principal's permission and the teachers' assistance. Participation was voluntary. Participants were explained that the data would be treated anonymously and confidentially, and only parents who had full-time jobs and worked for more than 30 h a week met the criterion for participation. Working parents and their children were kindly requested to complete the online questionnaire separately. The ethical approval of the study was given by the author's University Research Ethics Committee (Approval of IRB Protocol #2020-04-12e).

At Time 1, 986 adolescents rated the frequency of depressive moods they experienced over the last 2 weeks and their performance-avoidance orientation. Meanwhile, working parents rated their experiences of family-work conflict at the moment. Three weeks later, 468 parents rated their job engagement. The final matched samples consisted of 468 child-parent dyadic. Among adolescents, 44.7% were female, and the average age was 15.43 years (*SD* = 0.65). Among parents, 60.9% were female, and the average age was 42.86 years (*SD* = 5.66). Above 95% of parents who participated are married.

### 2.2. Measures

Survey items were back-translated according to the Brislin's (73) procedure. I used a response format of 1 = *strongly disagree* to 7 = *strongly agree* unless otherwise noted.

#### 2.2.1. Adolescent depression

Adolescents rated their depressive moods over the last 2 weeks with two items from the patient health questionnaire-4 (PHQ-4) scale (74). The scale begins with the stem question: “Over the last 2 weeks, how often have you been bothered by the following problems?” Response option is “not at all,” “several days,” “more than half the days,” and “nearly every day,” scored as 0, 1, 2, and 3, respectively. The scale assesses the frequency of core symptoms of depression, which are “Little interest or pleasure in doing things” and “Feeling down, depressed, or hopeless.” α = 0.80. In the present study, I used PHQ-2 scale to measure depressive moods (over 2 weeks). Although containing only 2 item, the PHQ-2 is widely used to assess depression in the general population (12, 75, 76). It is proved for having reliability and validity in the general population, and can be used to compare a subject's scale score with those determined from a general population reference group (74, 77, 78).

#### 2.2.2. Adolescent performance-avoidance

Adolescents reported performance-avoidance orientation using (32) three-item measure (e.g., “My goal in this class is to avoid performing poorly”; α = 0.77).

#### 2.2.3. Parent family-work conflict

At Time 2, parents rated their family-work conflict with a four-item scale (79). The sample item is “My personal demands are so great that it takes away from my work”; α = 0.77.

#### 2.2.4. Parent work engagement

Parents rated their work engagement using a nine-item scale adapted from Schaufeli et al.'s (69) scale (e.g., “At my work, I feel bursting with energy”; α = 0.95). All items are scored on a 5-point frequency rating scale ranging from 1 (never) to 5 (always).

#### 2.2.5. Control variables

Previous evidence suggests that children's clinical depression is more likely to influence mothers' clinical well-being (14, 35, 80). Also, compared to men, women are more likely to be affected by family members' momentary negative moods (81, 82). Moreover, there are gender differences in the effect of negative emotions at the work domain on one's home domain (83). Thus, parent gender was controlled. Also, adolescent gender affects parent-child communication of emotions, such that parents tend to be more emotional with the daughter than the son (84). Thus, adolescents' gender was also controlled. In addition, physical contiguity might be a condition for crossover to happen (81, 85). Therefore, whether the children lived in a dormitory was controlled.

## 3. Results

Table 1 shows the means, standard deviations, and intercorrelations.

**Table 1:** Descriptive statistics and correlations among study variables.

**Variable name**	**Mean**	***SD***	**1**	**2**	**3**	**4**	**5**	**6**	**7**
1. Adolescent gender	1.45	0.50	–						
2. Board at school	1.99	0.12	0.00	–					
3. Parent gender	1.61	0.49	0.09^*^	0.08	–				
4. Adolescent depressive moods	1.79	0.68	0.11^*^	0.05	0.03	(0.80)			
5. Adolescent performance–avoidance	4.22	1.56	0.05	−0.05	−0.01	0.23^**^	(0.77)		
6. Parent family–work conflict	2.70	1.32	0.05	−0.05	−0.04	0.19^**^	0.21^**^	(0.77)	
7. Parent job engagement	3.54	0.72	0.03	0.12^**^	0.06	−0.12^*^	−0.10^*^	−0.21^**^	(0.95)

*N* = 468 adolescent–parent dyadic. Adolescent gender and parent gender were coded as 1 = male, 2 = female. Board at school was coded as 1 = board at school, 2 = board at home.

^*^p < 0.05.

^**^p < 0.01.

Before testing the hypothesis, I conducted the confirmatory factor analysis using Mplus 7.4 with ML estimation to determine the distinctiveness of variables (i.e., adolescent depressive moods, adolescent performance-avoidance orientation, parents' family-work conflict, and parents' work engagement). As shown in Table 2, the hypothesized four-factor model was a better fit to data, χ^2^(129) = 478.83, *p* < 0.000, CFI = 0.93, TLI = 0.92, RMSEA = 0.08, SRMR = 0.04, than alternative models.

**Table 2:** Results of confirmatory factor analysis of study variables.

**Model**	**χ2**	***df***	***Δχ* ^2^**	**Δ*df***	**CFI**	**TLI**	**RMSEA**	**SRMR**
1.4–factor	478.83	129			0.93	0.92	0.08	0.04
2.3–factor	829.21	132	350.38	3	0.87	0.84	0.11	0.07
3.2–factor	1297.77	134	818.94	5	0.77	0.74	0.14	0.11
4.1–factor	1631.92	135	1153.09	6	0.71	0.67	0.15	0.13

df, degrees of freedom; Δχ^2^, chi–square differences; Δdf, degrees of freedom differences; CFI, Comparative Fit Index; TLI, Tucker–Lewis Index; RMSEA, Root Mean Square Error of Approximation; SRMR, Standardized Root Mean Square Residual. *N* = 468 adolescent–parent dyadic. Model 1 (4–factor model) includes all study variables (adolescents depression, adolescent performance–avoidance, parent family–work conflict, and parent job engagement) being treated as four independent factors. Model 2 (3–factor model) combine adolescent depression and performance–avoidance as one factor and treated the other two variables as two independent factors. Model 3 (2–factor model) combing adolescent depression and performance–avoidance as one factor, and the other two parent variables (family–work conflict and job engagement) as one factor. Model 4 (1–factor model) combine all four variables as one factor. Δχ^2^ and Δdf reflect differences of χ^2^ and df between the corresponding model and Model 1. All χ^2s^ and Δχ^2s^ are significant at *p* < 0.001 level.

All the hypotheses were tested in SPSS 26. As summarized in Table 3, after including the control variables, adolescent depressive moods were positively related to parents' family-work conflict (*b* = 0.37, *p* < 0.001), therefore hypothesis 1 was supported.

**Table 3:** Summary of regression analysis results of hypothesis 1.

**Variables**	**Parent family–work conflict (T2)**		**Parental work engagement (T3)**	
	***B***	***SE***	***B***	***SE***
Constant	3.30^**^	1.01	2.46^***^	0.54
**Controls**				
Adolescents gender	0.07	0.12	0.07	0.07
Board at school	−0.59	0.50	0.68^**^	0.27
Parent gender	−0.11	0.12	0.06	0.07
**Independent variable**				
Adolescents' depressive moods (T1)	0.37^***^	0.09	−0.10^*^	0.05
**Mediators**				
Parent family–work conflicts (T2)			−0.10^***^	0.03
*R* ^2^	4.90^***^	0.04	6.94^***^	0.07

*N* = 468 adolescent–parent dyadic. Adolescent gender and parent gender were coded as 1 = male, 2 = female. Board at school was coded as 1 = board at school, 2 = board at home.

^*^p < 0.05.

^**^p < 0.01.

^***^p < 0.001.

**Figure 2:**
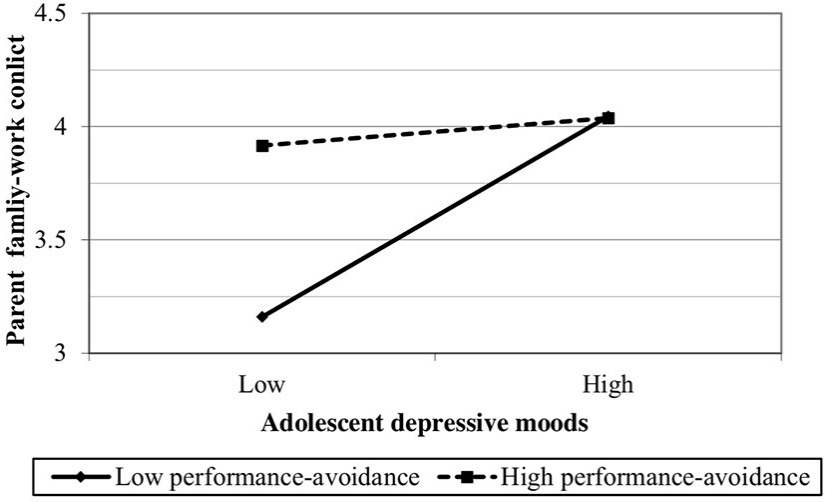
The effect of adolescents' depressive moods on parent family work conflict at high and low levels on adolescent performance-avoidance.

As shown in Table 4, there was a negative interaction between adolescent depressive moos and performance-avoidance orientation on parent family-work conflict (*b* = −0.18, *p* < 0.01). The relationship between adolescent depressive moods and parent family-work conflict at high and low levels of performance-avoidance (1 *SD* above and below the mean) was plotted, see Figure 2. The simple slope tests showed that the relationship between adolescent depressive moods and parent family-work conflict was more positive when adolescent's performance-avoidance is low (*b* = 0.65, *p* < 0.001) than that when adolescent's performance-avoidance is high (*b* = 0.09, *ns*), supporting Hypothesis 2.

**Table 4:** Summary of regression analysis of hypotheses 2–4.

**Variables**	**Parent family–work conflict**		**Parental work engagement**	
	***B***	***SE***	***B***	***SE***
Constant	3.79^***^	0.98	2.28^***^	0.55
**Controls**				
Adolescent gender	0.07	0.12	0.07	0.07
Board at school	−0.49	0.49	0.68^**^	0.27
Parent gender	−0.10	0.12	0.06	0.07
**Independent variable**				
Adolescent depressive moods	0.37^***^	0.09	−0.10^*^	0.05
**Moderator**				
Adolescent performance–avoidance	0.12^**^	0.04		
**Interaction term**				
Adolescent depressive moods × performance–avoidance	−0.18^**^	0.06		
**Mediators**				
Parent family–work conflict			−0.10^***^	0.03
*R^2^*	7.26^***^	0.09	6.94^***^	0.07

*N* = 468 adolescent–parent dyadic. Adolescent gender and parent gender were coded as 1 = male, 2 = female. Board at school was coded as 1 = board at school, 2 = board at home.

^*^p < 0.05.

^**^p < 0.01.

^***^p < 0.001.

Furthermore, as shown in Table 4, parent family-work conflict was negatively related to parent work engagement (*b* = −0.10, *p* < 0.001). Thus, Hypothesis 3 was supported. For Hypothesis 4, I ran process *via* SPSS 26 to test the conditional indirect effect of adolescent depressive moods on parental work engagement *via* parental family-work conflict under high and low adolescent performance-avoidance (see Table 5). The indirect effect of adolescent depressive moods on parental work engagement *via* parent family-work conflict was more positive when adolescent performance-avoidance is low (*b* = −0.07, 95% bias-corrected confidence interval [CI] = [−0.13, −0.03], excluding zero) than that when adolescent performance-avoidance is high (*b* = −0.01, 95% bias-corrected confidence interval [CI] = [−0.04, 0.02], including zero). Thus, hypothesis 4 was supported.

**Table 5:** Summary of moderated mediation analysis.

**Parent work engagement**	***Effect***	***SE***	***(Boot) LLCI***	***(Boot) ULCI***
M−1SD	−0.07	0.02	−0.13	−0.03
M	−0.04	0.01	−0.07	−0.02
M+1SD	−0.01	0.01	−0.04	0.02

*N* = 468 adolescent–parent dyadic.

## 4. Discussion

The antecedents of employees' work engagement have long been a hot topic that attracts much scholarly attention. In this research, I identify adolescent emotional experiences as one critical antecedent of employees' work engagement. Using multi-source, multi-wave dyadic data, I found that adolescents' performance-avoidance orientation moderates the indirect relationship between adolescents' depressive moods and parents' work engagement *via* increased parents' family-work conflict. Such that positive indirect relationship was stronger when performance-avoidance was high than when performance-avoidance was low. All the hypotheses were supported.

### 4.1. Theoretical contributions and future research

One key contribution of the study is to point out that children's persistent state-based moods influence working parents' family-work interface. Although parents are generally considered as the one who exerts influence in the parent-child relationship (e.g., transfer of momentary moods), I found that adolescents' depressive moods over the last 2 weeks have a non-egligible impact on parents' family-work conflicts. Though received relatively little attention, the child-parent level is a critical observational level for emotions and work-family interaction (30). Most of the research on the child-parent level holds that parents' emotional experience can transmit to their children, whereas children's emotions seldom reversely act on their parents (27). As more mature and powerful, parents might not be easily affected by children's momentary moods. However, working parents not only need to play a proper family role but also perform their work role. Children's persistent state-based moods, such as depressive moods, may cause parents to inevitably distribute more resources into their family role so that their input in work role is constrained. To be specific, I found that adolescent depressive moods lead to lower parents' work engagement by promoting a higher level of parents' family-work conflict. This study enriches the understanding and provides new insights into the role of adolescents' emotions in employees' family-work conflicts and work engagement.

Although many studies have examined the relationship between children's clinical depression and parental mental health, there is still relatively little research on short-term depressive moods in adolescents and the changes it causes in the family environment. The authors suggest that there are at least two reasons why researchers should further examine short-term depression in adolescents: (1) short-term depression is more prevalent than clinical depression; and (2) adolescence is a period of high depression prevalence, and the persistence of depression is an important influence on the development of depression. Research on depressive mood in early adolescence is beneficial in exploring how parental health and children's mental health develop in concert from a more microscopic perspective. This will provide a foundation for effective interventions.

The critical role of adolescent trait-based affect and its consequences on parent's family-work interaction are highlighted in the research. Trait-based affect, such as neuroticism, extraversion, agreeableness, and attachment style, are relevant to the work-family interface (30). Building further on this foundation, I emphasized the role of adolescent achievement goal orientation as a trait-based affect and its inter-individual, inter-domain effect on parents' work engagement for the first time. It shows that adolescents negative achievement goals will have negative impacts not only on themselves but also on their parents' work engagement. The research on achievement goals can focus not only on its consequences on the intra-individual, inter-domain level but also on the inter-individual, inter-domain level.

Furthermore, I also contribute to the literature by revealing the joint influence of adolescent depressive moods (persistent state-based moods) and performance-avoidance (trait-based affect) on parents' family-work interaction and work engagement. The duration of depressive moods might last 1 week to 6 months. During the process, parents' family-work conflict increases and their work engagement decreases. However, there is a boundary condition. When adolescents have a high level of performance-avoidance, their parents might constantly experience relatively higher levels of family-work conflict regardless of levels of children's depressive moods. However, when adolescents' performance-avoidance level is low, the positive relationship between adolescents' depressive moods and parents' family-work conflict is stronger. It points out one potential factor easily ignored to affect parents' family-work interaction: Adolescents' performance-avoidance orientation. Thus, the important, influential factors of employees' family-work conflict and work engagement are proposed.

### 4.2. Practical implications

Job engagement is the key driver of organizational success and competitive advantage (86, 87). Besides job characteristics, leadership, and dispositional characteristics (88), adolescents' emotional experiences may also impact parents' work engagement. Teenagers are the high-incidence group of depression (12–20%) and depressive moods (20–50%), which also means that their children's depressive moods threaten a considerable number of parents. It potentially impacts parents' work-life balance, organizations, and even society.

Being parents and employees at the same time, they should face up to negative feelings when dealing with their children's depressive moods. Much information guides parents on how to provide caregiving when their children are depressed instead of telling them it is normal that they are upset about it. Employees should actively guide and accept their psychological changes and needs timely adjust and actively intervene themselves. It is not clear whether higher parents' family-work conflicts will further hinder adolescents' emotional states. Such that parents' appropriate recognition and adjustment of their own situation may help to break the potential vicious circle between their children's depression and their working condition.

For organizations, I identified adolescent depressive moods as an important influencing factor for employees' work engagement, such that enterprises should take corresponding measures to deal with this challenge. Nowadays, many professional service firms demand to 24/7 availability (89, 90), which makes it more difficult to balance family and work for employees at the same time. In this condition, one's work role already constantly demands his/her extra energy to complete. The depressive moods of their children's might make it worse or even bring about a vicious circle of work-family interaction. In particular, women may face more severe challenges in 24/7 work culture (89). Therefore, gender issues should not be ignored. In other words, organizations should introduce more family-friendly policies and take the initiative to care for employees with teenagers. It is appropriate to offer some extra flexibility and welfare policies to the employees with depressed teenagers. In particular, more support can be given to working women.

### 4.3. Potential limitation and future research directions

Despite the contributions, there are some potential limitations which might offer promising directions for future research. First, because the study uses self-reported scales, it might not fully capture depressive moods as a complex psychological phenomenon, necessitating future research. Second, although the data were collected from multiple sources and time periods, the reverse causality could still be a possibility. For example, adolescent's depression could be influenced by parents' depression. Finally, I examine how parents, as influenced influencers, are influenced by adolescents' depression and achievement avoidance. However, in order to answer the question of the extent to which children's emotional experiences affect their parents work attitude and behavior, it is not enough to only study adolescent depression. More importantly, the results of the study are mainly valid for married/cohabiting parents and not separated/divorced parents.

## Data availability statement

The raw data supporting the conclusions of this article will be made available by the author, without undue reservation.

## Ethics statement

The studies involving human participants were reviewed and approved by Research Ethics Committee Peking University. Written informed consent to participate in this study was provided by the participants' legal guardian/next of kin.

## Author contributions

LL: conceptualization, methodology, formal analysis, writing-original draft, and writing-review and editing.
